# Advancing breastfeeding promotion: leveraging integrative natural galactagogues and unveiling their potential roles—Insights from a 19-year Taiwan nationwide registry to address lactation insufficiency in postpartum women

**DOI:** 10.3389/fnut.2024.1293735

**Published:** 2024-02-02

**Authors:** Chung-Chih Liao, Chi-Hsien Chien, Tzu-Ju Hsu, Jung-Miao Li

**Affiliations:** ^1^Department of Post-Baccalaureate Veterinary Medicine, College of Medical and Health Science, Asia University, Taichung, Taiwan; ^2^Chuyuan Chinese Medicine Clinic, Taichung, Taiwan; ^3^Department of Post-Baccalaureate Veterinary Medicine, Asia University, Taichung, Taiwan; ^4^Management Office for Health Data, China Medical University Hospital, Taichung, Taiwan; ^5^School of Chinese Medicine, College of Chinese Medicine, China Medical University, Taichung, Taiwan; ^6^Department of Chinese Medicine, China Medical University Hospital, Taichung, Taiwan

**Keywords:** lactation insufficiency, agalactia, hypogalactia, Chinese herbal products, prescription patterns, network analysis

## Abstract

**Background:**

Lactation insufficiency is a prevalent challenge for nursing mothers globally. There is a growing interest in the use of herbal galactagogues for enhancing lactation, but their therapeutic efficacy and underlying mechanisms need thorough investigation. This study aims to investigate the efficacy and mechanisms of action of herbal galactagogues in addressing lactation insufficiency by utilizing real-world data and employing a network analysis approach.

**Methods:**

Our retrospective study used Taiwan’s Longitudinal Health Insurance Database 2000 (LHID2000) to identify 490 patients diagnosed with lactation insufficiency from 2000 to 2018. We analyzed demographic characteristics, co-existing diseases, and prescription patterns for both users and non-users of Chinese herbal products (CHP). Additionally, we utilized a network analysis approach to explore potential compounds and targets in the most frequently used CHP, the Wang Bu Liu Xing and Lu Lu Tong herb pair (WLHP) combination.

**Results:**

Out of 490 patients, 81% were CHP users. There were no significant differences in demographic characteristics between CHP users and non-users, but we observed a notable divergence in the prevalence of co-existing diseases. A detailed examination of CHP prescriptions revealed the predominance of WLHP, prompting further investigation. Comprehensive analysis identified 29 major compounds in WLHP, which were associated with 215 unique targets. Intersection analysis revealed 101 overlapping targets between WLHP and lactation, suggesting their potential as therapeutic targets for lactation insufficiency treatment. Topological analysis of the protein-protein interaction (PPI) network identified 13 hub genes potentially crucial for the therapeutic effect of WLHP. Functional enrichment analysis showed that these targets were involved in critical lactation regulation pathways, including the PI3K-Akt signaling pathway, prolactin signaling pathway, estrogen signaling pathway, and AMPK signaling pathway.

**Discussion:**

This study emphasizes the potential of CHP, specifically the WLHP combination, in managing lactation insufficiency. The multi-compound, multi-target approach of WLHP and its interaction with key biological processes and signaling pathways offer valuable insights into the underlying mechanisms of its therapeutic effects. These findings warrant further experimental validation and can guide future research and clinical applications of CHP in lactation insufficiency treatment.

## 1 Introduction

Breast milk is a unique and irreplaceable nutritional source, offering essential macronutrients such as carbohydrates, proteins, and fats, alongside micronutrients like vitamins and minerals, specifically tailored to an infant’s needs. Additionally, the breast milk contains a diverse array of microorganisms, collectively known as the milk microbiota, whose interactions with the mother-child microbiome are essential for the infant’s health and play a pivotal role in their early development (1–4). It also contains biocompounds, including like secretory immunoglobulin A, growth factors, and hormones, which support the infant immune system and promote optimal development (5–7). Besides physical benefits, breastfeeding is vital to foster maternal-infant bonding and offers emotional and psychological advantages for both the mother and baby (8, 9).

Optimal lactation is essential for infant growth, development, and overall wellbeing (10). Lactation insufficiency, which includes conditions like agalactia (the complete absence of milk production) and hypogalactia (diminished milk production), is a significant concern for many postpartum women. It affects the health and wellbeing of both the mother and the infant (11, 12). Lactation insufficiency can result from various factors, including hormonal imbalances such as insufficient prolactin or oxytocin production (13), inadequate glandular tissue impairing milk synthesis (14), inadequate diet, and suboptimal breastfeeding practices like poor latching or infrequent feeding (14, 15). The use of galactagogues, substances that promote lactation, is a common approach to address lactation insufficiency (16). Pharmacological galactagogues, such as metoclopramide and domperidone, are extensively utilized in clinical practice (17). However, the potential side effects and contraindications associated with pharmacological galactagogues have raised concerns among healthcare professionals and patients. These issues include possible central nervous system side effects, cardiac arrhythmias, and drug interactions, posing risks to both the mother and infant (18, 19). Therefore, there is a growing need to explore alternative solutions, particularly herbal galactagogues, which are considered safer and more natural options for promoting lactation (20, 21).

Traditional Chinese medicine (TCM) has a long-standing and rich history of employing herbal galactagogues to address lactation insufficiency and enhance milk production (22). Herbal remedies, including galactagogues, are carefully selected and combined based on their synergistic properties to promote optimal lactation and overall wellbeing. Ancient TCM texts and empirical knowledge passed down through generations have documented various herbal galactagogues and their specific roles in supporting lactation. These herbs are believed to function through multiple mechanisms, such as unblocking meridians [“meridians” are conceptualized as a network of pathways in the body through which energy and information are thought to circulate (23)], nourishing the blood, tonifying Qi [“Qi” is a vital energy or life force that circulates throughout the body, crucial for health and wellbeing (24)], and promoting fluid production. Consequently, TCM herbal galactagogues offer a holistic approach to address lactation insufficiency, targeting the enhancement of milk production as well as improvement of maternal health and wellbeing. In Taiwan, TCM plays an integral role in healthcare, and the use of Chinese herbal products (CHP) for lactation support is widespread (22, 25). However, a thorough comprehension of their prescription habits remains to be achieved.

In this study, we aimed to uncover the prescription patterns and therapeutic potential of CHP in addressing lactation insufficiency in Taiwan. To accomplish this, we combined real-world evidence from the National Health Insurance Research Database (NHIRD) and network analysis to obtain a comprehensive perspective.

## 2 Materials and methods

### 2.1 Real world data source

The NHIRD is an extensive medical database created and maintained by the Taiwan National Health Insurance administration. It encompasses a wide range of longitudinal data on healthcare services provided to insured individuals, including demographic information, diagnoses, treatment procedures, and prescribed medications. The NHIRD serves as an invaluable asset for researchers conducting large-scale population-based studies across various medical fields, such as epidemiology, healthcare utilization, and drug safety (26). The NHIRD follows the International Classification of Diseases, 9th Revision, and 10th Revision, Clinical Modification (ICD-9-CM and ICD-10-CM, respectively) for disease coding. This study was approved by the Institutional Review Board of the Research Ethics Committee of China Medical University Hospital (CMUH111-REC2-109).

### 2.2 Study population

**FIGURE 1:**
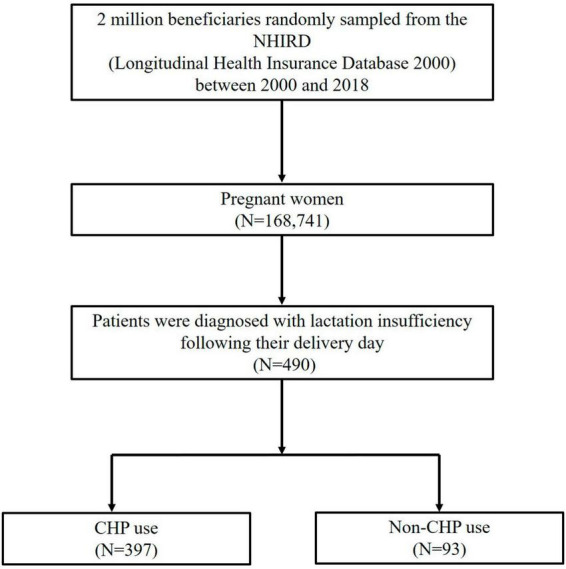
Flow chart depicting the study design and patient selection process for lactation insufficiency.

This retrospective observational population-based study used the Longitudinal Health Insurance Database 2000 (LHID2000) to select patients from January 1, 2000, to December 31, 2018. The flow chart is shown in Figure 1. Initially, we identified pregnant women (ICD9-V22, V23, V27.0, V27.2, V27.3, V27.5, V27.6, V30-V39, 650; ICD10-O09, O36.8, O80, O99.84, Z32.01, Z33.1, Z34, Z37.0, Z37.2, Z37.3, Z37.5, Z37.6, Z38) within the LHID2000 (*n* = 168,741). Following delivery, lactation insufficiency (ICD9-676.4, 676.84; ICD10-O92.3, O92.4) was identified in patients with diagnostic records (*n* = 490). Individuals who visited a Chinese medicine clinic and received CHP treatment at least once within 1 month after being diagnosed with lactation insufficiency were defined as “CHP users,” while those who did not use CHP were considered “non-CHP users.” The decision to classify “CHP users” in this manner was based on two main considerations: (1) the timeliness of treatment, as the first month after diagnosis is a critical period for addressing lactation insufficiency and providing timely intervention is essential to improve the health of both the mother and infant, and (2) treatment adherence, as a 1-month time frame helps identify patients who actively sought and adhered to CHP treatment for lactation insufficiency, allowing the study to better assess the prescription patterns of CHP treatment in the target population.

### 2.3 Assessment of covariates

The sociodemographic factors assessed included age, income, and urbanization level. Adult patients were further divided into three subgroups by age: 18–30, 31–40, and 41–50 years. Income, determined by individual working salary, was classified into three levels: <20,000, 20,000–39,999, and ≥40,000 NT$/month. Additionally, the urbanization level of townships in Taiwan was categorized based on the population characteristics including educational level, density, proportion of elderly individuals, and general occupation, ranging from the highest to lowest across the three levels. We also collected data on commonly prescribed medications related to lactation insufficiency, such as pharmacological galactagogues, including metoclopramide (ATC code-A03FA01), domperidone (ATC code-A03FA03), and sulpiride (ATC code-N05AL01).

### 2.4 Analysis of co-existing diseases and CHP use in lactation insufficiency

We examined the frequency distribution of co-existing diseases in both CHP users and non-CHP users following a diagnosis of lactation insufficiency, based on ICD-9 and ICD-10 codes. Subsequently, for the CHP user group, we examined the prescribed treatments, including calculation of the frequency of single Chinese herbs, Chinese herbal formulas, and dual CHP combinations specifically used for lactation insufficiency. To gain insight into the relationships between different herbs and herbal formulas used for lactation insufficiency and to identify a core prescription pattern, we conducted a correlation network analysis of the common CHP combinations.

### 2.5 Statistical analysis

Categorical variables are presented by sample size, with the chi-square test used to determine differences between CHP and non-CHP users. Continuous variables are presented as mean and standard deviation, with *t*-tests used to identify differences. The odds ratios of the variables were calculated using a logistic regression model, adjusting for age group, income, urbanization, and medications. All statistical analyses were performed using SAS software, version 9.4 (SAS Institute Inc., Cary, NC), and the correlation network graph of common CHP combinations was created using RStudio.

### 2.6 Herbal compound collection and target identification for Wang Bu Liu Xing [*Vaccaria segetalis* (Neck.) garcke] and Lu Lu Tong (*Liquidambar formosana* hance) herb pair (WLHP)

A comprehensive search for the chemical constituents of WLHP, comprising Wang Bu Liu Xing and Lu Lu Tong, was conducted using the Traditional Chinese Medicine Systems Pharmacology (TCMSP) database.^1^ Our study included all identified compounds, without filtering based on oral bioavailability (OB) or drug-likeness (DL), to thoroughly explore the potential therapeutic effects of these herbs. The compound targets were retrieved from the TCMSP database and mapped to their gene symbols using the UniProt database^2^ for further analysis.

### 2.7 Lactation-related target screening and correlative target identification between WLHP and lactation

A comprehensive list of lactation-related targets was compiled through a systematic search in the Online Mendelian Inheritance in Man (OMIM),^3^ DisGeNET,^4^ and GeneCards^5^ databases using the keyword “lactation” for “*Homo sapiens*.” After eliminating duplicates and invalid targets, the remaining targets were used to establish connections between WLHP and lactation. Overlapping WLHP and lactation targets were considered correlative targets for treating lactation insufficiency. The Venn online tool^6^ was used for intersection analysis.

### 2.8 PPI network construction

Protein-protein interaction networks for intersecting gene targets were constructed using the STRING database.^7^ The networks were limited to “*Homo sapiens*” with a combined interaction score of ≥0.7 (high confidence) and were visualized and analyzed using Cytoscape software (version 3.9.1) to understand the target relationships and interactions.

### 2.9 Network topology analysis and hub gene identification

The CytoHubba plugin in Cytoscape was used to analyze the PPI network topology and identify hub genes. Various topology algorithms, including maximum neighborhood component (MNC), edge percolated component (EPC), degree, betweenness, and closeness, were applied to the rank nodes. Nodes with high rankings in multiple algorithms were considered potential hub genes, playing crucial roles in the therapeutic effects of WLHP on lactation insufficiency.

### 2.10 Functional enrichment analysis

To understand the biological functions and pathways associated with overlapping targets between WLHP and lactation, functional enrichment analysis was performed using Metascape.^8^ Gene Ontology (GO) terms for biological process (BP), cellular component (CC), and molecular function (MF), as well as Kyoto Encyclopedia of Genes and Genomes (KEGG) pathways, were enriched. A *p*-value < 0.01 was set as the threshold for statistical significance.

## 3 Results

### 3.1 Demographic characteristics and pharmacological galactagogue use among CHP users and non-CHP users with lactation insufficiency

In total, 490 patients diagnosed with lactation insufficiency were identified in the LHID2000 from 2000 to 2018. Of these, 397 were categorized as CHP users (81%), and 93 as non-CHP users (19%). Table 1 presents the demographic characteristics and medication use in both groups. The average age of the study population was approximately 33 years. Most patients with lactation insufficiency in both groups belonged to the 31–40-year age bracket, earned less than 20,000 NT$ per month, and were from the most urbanized areas. A comparative analysis between CHP users and non-CHP users revealed no significant disparities in terms of age, income, and urbanization level (*p* > 0.05). In the context of pharmacological galactagogue usage, domperidone was the most commonly prescribed medication among both CHP and non-CHP users. Specifically, it was administered to 83.4% of CHP users and 68.8% of non-CHP users, a statistically significant difference (*p* = 0.0014). Metoclopramide was also widely used and was administered to 70.5% of CHP users and 67.7% of non-CHP users. However, the difference was not statistically significant (*p* = 0.5976). Sulpiride was less frequently prescribed, to 12.8% of CHP users and 10.8% of non-CHP users, with no significant difference noted between the two groups (*p* = 0.5820).

**TABLE 1:** Characteristics of Chinese herbal product (CHP) and non-CHP users with lactation insufficiency.

Variable	Chinese herbal product				*P-*value
	No (*n* = 93)		Yes (*n* = 397)		
	*n*	%	*n*	%	
Age					0.0821
18–30	33	35.5	96	24.2	
31–40	57	61.3	284	71.5	
41–50	3	3.2	17	4.3	
Mean (SD)	31.53 (4.79)		33.06 (4.05)		0.0051
Income amount (NT$/month)^†^					0.7098
<20,000	54	58.0	244	61.4	
20,000–39,999	30	32.3	111	28.0	
> = 40,000	9	9.7	42	10.6	
Urbanization^‡^					0.1282
1 (highest)	46	49.5	237	59.7	
2	40	43.0	127	32.0	
3 (lowest)	7	7.5	33	8.3	
Pharmacological galactagogue					
Metoclopramide	63	67.7	280	70.5	0.5976
Domperidone	64	68.8	331	83.4	0.0014
Sulpiride	10	10.8	51	12.8	0.5820

^†^New Taiwan Dollar (NTD), 1 NTD is equal to 0.03 USD. ^‡^Urbanization level was divided by the population density of the residential area into three levels, where level 1 was the most urbanized and level 3 was the least urbanized.

### 3.2 Co-existing diseases in CHP and non-CHP users with lactation insufficiency

Table 2 presents the frequency distribution of co-existing diseases in both the CHP and non-CHP user groups. The range of co-existing diseases in CHP users was more diverse than that in non-CHP users, including conditions such as myalgia, dyspepsia, sleep disturbances, lumbago, the common cold, headache, and constipation. Conversely, in non-CHP users, coexisting diseases were predominantly related to musculoskeletal and joint pain.

**TABLE 2:** Co-existing diseases in patients with lactation insufficiency between Chinese herbal product (CHP) users and non-CHP users.

Rank	Non-CHP users	*n*	CHP users	n
1	719.4 (Pain in joint)	9	729.1 (Myalgia and myositis)	141
2	729.1 (Myalgia and myositis)	7	536.8 (Dyspepsia and other specified disorders of function of stomach)	127
3	727.0 (synovitis and tenosynovitis)	5	780.5 (Sleep disturbances)	92
4	723.3 (Cervicobrachial syndrome)	4	724.2 (Lumbago)	92
5	847.2 (Sprains and strains of lumbar)	4	460 [Acute nasopharyngitis (common cold)]	91
6	922.3 (Contusion of back)	3	784.0 (Headache)	76
7	924.1 (Contusion of lower leg)	3	564.0 (Constipation)	72
8	S602 (Contusion)	3	786.2 (Cough)	60

### 3.3 Prescription patterns of single Chinese herbs and Chinese herbal formulas for lactation insufficiency

Tables 3, 4 present the top 10 most frequently prescribed single Chinese herbs and herbal formulas for lactation insufficiency, along with their average daily dose and traditional pharmacological actions. The three most frequently prescribed single Chinese herbs were Wang Bu Liu Xing (407 prescriptions), Lu Lu Tong (215 prescriptions), and Du Zhong (130 prescriptions). Jia Wei Xiao Yao San (prescribed 155 times), Ba Zhen Tang (prescribed 49 times), and Gui Pi Tang (prescribed 48 times) were the most commonly used Chinese herbal formulas.

**TABLE 3:** Top 10 most frequently prescribed single Chinese herbs for lactation insufficiency.

Single Chinese herbs	Botanical name	English name	Traditional pharmacological action	Frequency	Average daily dose (g)	Average duration of prescription (days/visit)
Wang Bu Liu Xing	*Vaccaria segetalis* (Neck.) Garcke	Cowherb Seed	Invigorates blood, reduces swelling, promotes lactation, and clears blood stasis	407	1.6	7.2
Lu Lu Tong	*Liquidambar formosana* Hance	Beautiful Sweetgum Fruit	Promotes blood circulation, unblocks channels, relieves pain, and alleviates joint stiffness	215	1.2	7.0
Du Zhong	*Eucommia ulmoides* Oliv.	Eucommia Bark	Tonifies the liver and kidney, strengthens bones and sinews, and lowers blood pressure	130	1.1	7.6
Huang Qi	*Astragalus membranaceus* (Fisch.) Bunge	Astragalus Root	Tonifies Qi, augments the spleen, raises Yang Qi, and enhances immune function	124	4.0	7.2
Tong Cao	*Tetrapanax papyrifer* (Hook.) K.Koch	Rice Paper Plant Pith	Promotes urination, clears heat, and expels phlegm	123	1.3	7.0
Si Gua Luo	*Luffa cylindrica* (L.) Roem.	Loofah, Towel Gourd Vegetable Sponge	Expels wind, clears heat, promotes diuresis, and unblocks channels	82	1.1	7.3
Dang Gui	*Angelica sinensis* (Oliv.) Diels	Chinese Angelica Root	Tonifies blood, regulates menstruation, alleviates pain, and moistens the intestines	67	3.9	7.2
Pu Gong Ying	*Taraxacum mongolicum Hand.-Mazz.*	Mongolian Dandelion Herb	Clears heat, eliminates toxins, reduces swelling, and promotes lactation	66	1.3	6.8
Tu Si Zi	*Cuscuta chinensis* Lam.	Chinese Dodder Seed	Tonifies the kidney, nourishes the liver, improves vision, and enhances fertility	65	1.1	7.4
Qing Pi	*Citrus reticulata* Blanco	Green Tangerine Peel	Regulates Qi, soothes the liver, breaks Qi stagnation, and relieves pain	60	0.9	6.9

**TABLE 4:** Top 10 most frequently prescribed Chinese herbal formulas for lactation insufficiency.

Chinese herbal formulae	Ingredients	Traditional pharmacological action	Frequency	Average daily dose (g)	Average duration of prescription (days)
Jia Wei Xiao Yao San	Chai Hu (*Bupleurum chinense* DC.), Dang Gui [*Angelica sinensis* (Oliv.) Diels], Bai Shao (*Paeonia lactiflora* Pall.), Bai Zhu (*Atractylodes macrocephala* Koidz.), Fu Ling [*Wolfiporia extensa* (Peck) Ginns], Mu Dan Pi (*Paeonia suffruticosa* Andr.), Zhi Zi (*Gardenia jasminoides* J.Ellis), Gan Cao (*Glycyrrhiza uralensis* Fisch.), Bo He (*Mentha haplocalyx* Briq.), Sheng Jiang (*Zingiber officinale* Roscoe)	Alleviates stress, soothes liver Qi, and addresses conditions like depression, irritability, and menstrual irregularities.	155	4.4	7.0
Ba Zhen Tang	Ren Shen (*Panax ginseng* C.A.Mey.), Bai Zhu (*Atractylodes macrocephala* Koidz.), Fu Ling [*Wolfiporia extensa* (Peck) Ginns], Zhi Gan Cao (*Glycyrrhiza uralensis* Fisch), Shu Di Huang [*Rehmannia glutinosa* (Gaertn.) DC. Praeparata], Bai Shao (*Paeonia lactiflora* Pall.), Dang Gui [*Angelica sinensis* (Oliv.) Diels], Chuan Xiong (*Ligusticum chuanxiong* Hort)	Strengthens Qi and nourishes blood, alleviates fatigue, weakness, and pale complexion	49	6.3	7.2
Gui Pi Tang	Ren Shen (*Panax ginseng* C.A.Mey.), Huang Qi [*Astragalus membranaceus* (Fisch.) Bunge], Bai Zhu (*Atractylodes macrocephala* Koidz.), Fu Ling (*Wolfiporia extensa* (Peck) Ginns), Suan Zao Ren (*Ziziphus jujuba* var. spinosa (Bunge) Hu ex H.F.Chou), Long Yan Rou (*Dimocarpus longan* Lour.), Mu Xiang [*Saussurea costus* (Falc.) Lipsch.], Dang Gui [*Angelica sinensis* (Oliv.) Diels], Yuan Zhi (*Polygala tenuifolia* Willd.), Zhi Gan Cao (*Glycyrrhiza uralensis* Fisch.)	Tonifies spleen Qi, nourishes heart blood, and treats insomnia, anxiety, and poor memory	48	3.3	6.6
Si Wu Tang	Shu Di Huang (*Rehmannia glutinosa* (Gaertn.) DC. praeparata), Bai Shao (*Paeonia lactiflora* Pall.), Dang Gui [*Angelica sinensis* (Oliv.) Diels], Chuan Xiong (*Ligusticum chuanxiong* Hort).	Nourishes and tonifies blood, regulates menstruation, and relieves pain	47	4.3	9.1
Sheng Yu Tang	Shu Di Huang (*Rehmannia glutinosa* (Gaertn.) DC. praeparata), Dang Gui (*Angelica sinensis* (Oliv.) Diels), Chuan Xiong (*Ligusticum chuanxiong* Hort), Bai Shao (*Paeonia lactiflora* Pall.), Ren Shen (*Panax ginseng* C.A.Mey.), Huang Qi (*Astragalus membranaceus* (Fisch.) Bunge).	Promotes tissue regeneration, accelerates wound healing, and addresses post-surgical recovery and trauma	44	4.0	6.6
Chai Hu Shu Gan Tang	Chai Hu (*Bupleurum chinense* DC.), Xiang Fu (*Cyperus rotundus* L.), Bai Shao (*Paeonia lactiflora* Pall.), Zhi Ke (*Citrus aurantium* L.), Chuan Xiong (*Ligusticum chuanxiong* Hort), Gan Cao (*Glycyrrhiza uralensis* Fisch.), Chen Pi (*Citrus reticulata* Blanco)	Regulates liver Qi, alleviates pain, and addresses conditions like depression, irritability, and abdominal pain	35	2.9	6.8
Ma Zi Ren Wan	Ma Zi Ren (*Cannabis sativa* L.), Bai Shao (*Paeonia lactiflora* Pall.), Hou Po (*Magnolia officinalis* Rehder & E.H.Wilson), Xing Ren (*Prunus armeniaca* L.), Da Huang (*Rheum palmatum* L.), Zhi Shi (*Citrus aurantium* L.)	Moistens the intestines, unblocks the bowels, drains heat, and regulates the qi to alleviate constipation and pain	33	2.3	7.2
Gui Zhi Fu Ling Wan	Gui Zhi (*Cinnamomum cassia* Presl), Fu Ling [*Wolfiporia extensa* (Peck) Ginns], Chi Shao (*Paeonia lactiflora* Pall.), Mu Dan Pi (*Paeonia suffruticosa* Andr.), Tao Ren [*Prunus persica* (L.) Batsch]	Invigorates blood, dispels blood stasis, reduces masses, alleviates pain, and warms the meridians	32	3.2	6.3
Du Huo Ji Sheng Tang	Du Huo (*Angelica pubescens* Maxim.), Sang Ji Sheng [*Taxillus chinensis* (DC.) Danser], Qin Jiao (*Gentiana macrophylla* Pall.), Fang Feng [*Saposhnikovia divaricata* (Turcz.) Schischk.], Dang Gui [*Angelica sinensis* (Oliv.) Diels], Ren Shen (*Panax ginseng* C.A.Mey.), Fu Ling [*Wolfiporia extensa* (Peck) Ginns], Du Zhong (*Eucommia ulmoides* Oliv.), Niu Xi (*Achyranthes bidentata* Blume), Chuan Xiong (*Ligusticum chuanxiong* Hort), Gan Cao (*Glycyrrhiza uralensis* Fisch.), Rou Gui (*Cinnamomum cassia* Presl), Bai Shao (*Paeonia lactiflora* Pall.), Sheng Di Huang [*Rehmannia glutinosa* (Gaertn.) DC.]	Expels wind-dampness, tonifies the liver and kidneys, strengthens the sinews and bones, and alleviates pain	32	2.3	6.6
Ren Shen Yang Rong Tang	Ren Shen (*Panax ginseng* C.A.Mey.), Fu Ling [*Wolfiporia extensa* (Peck) Ginns], Bai Zhu (*Atractylodes macrocephala* Koidz.), Bai Shao (*Paeonia lactiflora* Pall.), Yuan Zhi (*Polygala tenuifolia* Willd.), Wu Wei Zi [*Schisandra chinensis* (Turcz.) Baill.], Dang Gui [*Angelica sinensis* (Oliv.) Diels], Huang Qi [*Astragalus membranaceus* (Fisch.) Bunge], Shu Di Huang [*Rehmannia glutinosa* (Gaertn.) DC. praeparata], Zhi Gan Cao (*Glycyrrhiza uralensis* Fisch.), Rou Gui (*Cinnamomum cassia* Presl), Chen Pi (*Citrus reticulata* Blanco), Sheng Jiang (*Zingiber officinale* Roscoe), Da Zao (*Ziziphus jujuba* Mill.).	Tonifies Qi, nourishes blood, and treats conditions like chronic fatigue, dizziness, and palpitations	30	5.0	6.7

### 3.4 Analysis of common CHP combinations and the correlation network for lactation insufficiency

Table 5 lists the top 10 most frequently prescribed dual combinations of CHP for lactation insufficiency. The most frequent dual combinations were Wang Bu Liu Xing plus Lu Lu Tong, Jia Wei Xiao Yao San plus Lu Lu Tong, and Du Zhong plus Lu Lu Tong.

**TABLE 5:** Top 10 most frequently prescribed dual combinations of Chinese herbal products (CHP) for lactation insufficiency.

Rank	CHP name 1	CHP name 2	Number of prescriptions
1	Wang Bu Liu Xing	Lu Lu Tong	76
2	Jia Wei Xiao Yao San	Lu Lu Tong	68
3	Du Zhong	Lu Lu Tong	60
4	Qing Pi	Lu Lu Tong	48
5	Jia Wei Xiao Yao San	Du Zhong	47
6	Jia Wei Xiao Yao San	Qing Pi	46
7	Wang Bu Liu Xing	Tong Cao	42
	Jia Wei Xiao Yao San	Tu Si Zi	42
	Du Zhong	Qing Pi	42
10	Tu Si Zi	Lu Lu Tong	41

**FIGURE 2:**
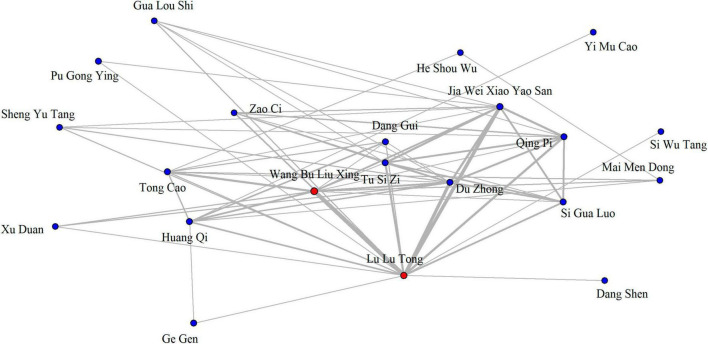
Correlation network diagram illustrating the interrelationship between different Chinese herbs and herbal formulas used for lactation insufficiency.

The correlation network graph, as illustrated in Figure 2, is a visual representation of the relationships between various Chinese herbs and herbal formulas used to treat lactation insufficiency. This network graph was constructed by analyzing the 66 most commonly prescribed CHP combinations (those prescribed more than 15 times) using RStudio. In the graph, each node represents an herb or herbal formula, and the edges (connecting lines) signify their co-prescription for treating lactation insufficiency. The thickness of each edge reflects the strength of the correlation or frequency of the co-prescription between two nodes, with thicker edges indicating a stronger correlation or higher frequency. In our study, the pairing of Wang Bu Liu Xing and Lu Lu Tong herbs, also known as WLHP, was identified as the most frequently occurring combination. This was determined by identifying the nodes with the most connections (indicating that they were commonly paired with other herbs or formulas) and the thickest edges (signifying a high frequency of co-prescription).

Notably, the prominence of the WLHP combination in this network indicates its critical role in treating lactation insufficiency according to the traditional Chinese medicinal approach. Continuing our focus on this intriguing result, we further explored the potential mechanisms underlying the efficacy of WLHP in addressing lactation insufficiency.

### 3.5 Identification of compounds and targets in WLHP

To gain a thorough understanding of the compounds and their associated targets in WLHP, we conducted a comprehensive search of the TCMSP database. This search revealed 42 compounds: 26 in Wang Bu Liu Xing and 16 in Lu Lu Tong. After removing compounds without known targets, we obtained 18 main compounds from Wang Bu Liu Xing and 11 from Lu Lu Tong. Using the TCMSP and UniProt databases, 215 unique targets associated with these compounds were retrieved for further analysis.

### 3.6 Identification of lactation-related targets and their correlation with WLHP

**FIGURE 3:**
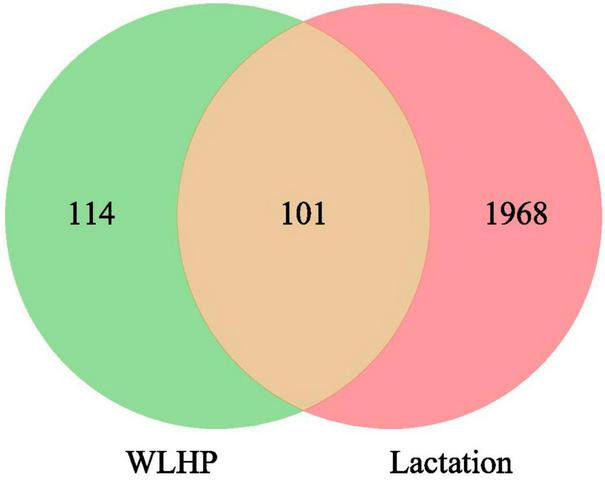
Venn diagram illustrating the overlap of therapeutic targets between WLHP and lactation-related targets.

To explore the potential therapeutic effects of WLHP on lactation insufficiency, identifying overlapping targets between WLHP and lactation is essential. We performed a systematic search of the OMIM, DisGeNET, and GeneCards databases to identify lactation-related targets. After removing duplicates and invalid targets, 2,069 lactation-related targets remained. Intersection analysis revealed 101 overlapping targets between WLHP and lactation, indicating their potential as therapeutic targets for lactation insufficiency (Figure 3).

### 3.7 Construction of the “Chinese Herbs-Components-Targets” network

**FIGURE 4:**
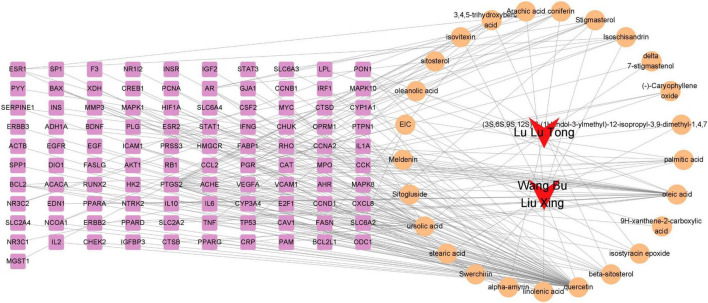
Comprehensive “Chinese herbs-components-targets” network depicting the complex interactions between WLHP and lactation.

To explore the complex relationship between Chinese herbs, compounds, and common targets, we identified 101 WLHP-lactation overlapping targets regulated by 25 WLHP compounds. Among these, 89 targets were regulated by 14 compounds from Wang Bu Liu Xing, and 33 targets were regulated by 11 compounds from Lu Lu Tong, with 21 shared targets. We used Cytoscape software to construct the “Chinese Herbs-Components-Targets” network, visualizing the intricate interactions between WLHP and lactation. The network comprised 128 nodes and 201 edges, with red nodes representing Chinese herbs, yellow nodes representing components, and pink nodes representing targets (Figure 4). This network demonstrates the multi-compound, multi-target nature of WLHP, suggesting that its effect on lactation is a complex regulatory process. The top three components with the highest connectivity were quercetin (found in Wang Bu Liu Xing), oleic acid (found in Wang Bu Liu Xing), and ursolic acid (Lu Lu Lu Tong), with degree values of 62, 25, and 21, respectively. These components are considered crucial for the therapeutic effect of WLHP on lactation insufficiency.

### 3.8 Analysis of PPI networks for WLHP-lactation overlapping targets

**FIGURE 5:**
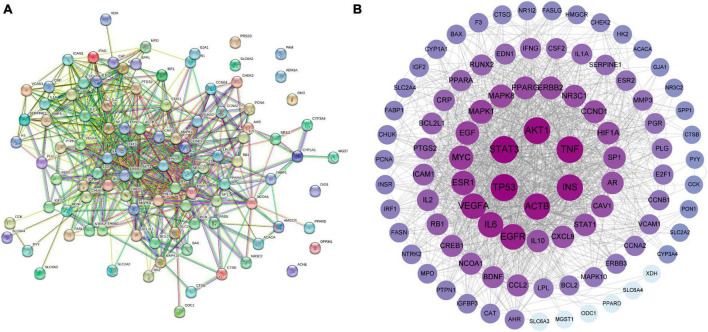
Protein-protein interaction (PPI) network analysis of overlapping targets between WLHP and lactation **(A)** Visualization of the PPI network constructed using the STRING database. **(B)** PPI network analysis using Cytoscape software, illustrating the intricate interactions among overlapping targets. Nodes represent targets and edges represent interactions. Node size and color intensity indicate the strength of target interactions.

To better understand the interactions between the 101 overlapping targets, we constructed a PPI network using the STRING database and Cytoscape software. The network contained 101 nodes (targets) and 658 edges (interactions), with larger and darker nodes representing stronger target interactions (Figures 5A, B). The network exhibited an average node degree of 13 and a PPI enrichment *p*-value < 1.0e-16, indicating a significantly higher number of interactions than that expected for a random set of proteins. The high number of interactions observed in the PPI network suggests that these targets may have a coordinated regulatory role in WLHP-lactation. This further supports the notion that WLHP affects lactation in a complex and interconnected manner.

### 3.9 Identification of hub genes from PPI network analysis

**FIGURE 6:**
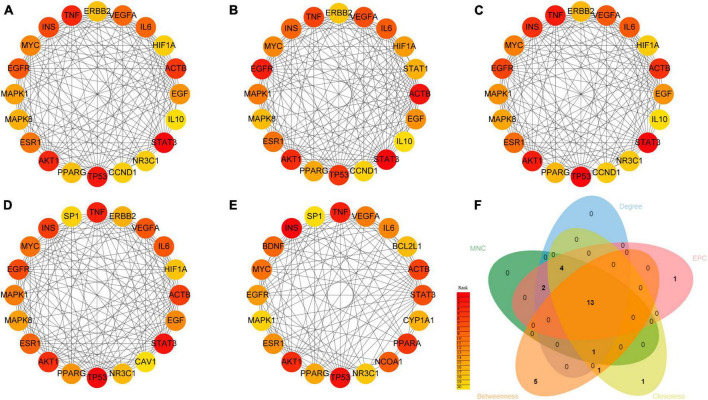
Hub gene analysis from the protein-protein interaction (PPI) network. **(A)** Top 20 hub genes identified based on the maximum neighborhood component (MNC). **(B)** Top 20 hub genes identified based on the edge percolated component (EPC). **(C)** Top 20 hub genes identified based on degree. **(D)** Top 20 hub genes identified based on betweenness. **(E)** Top 20 hub genes identified based on closeness. **(F)** Intersection of Top 20 hub genes from five different topological algorithms.

To identify the key genes potentially involved in the therapeutic effects of WLHP on lactation insufficiency, we used the CytoHubba plugin in Cytoscape to analyze the PPI network. This revealed the top 20 target genes based on five forms of topological properties (Figures 6A–E). We then performed a Venn analysis across these five topology algorithms, which identified 13 potential hub genes (Figure 6F). These hub genes, including STAT3, TP53, AKT1, TNF, ACTB, INS, EGFR, IL6, VEGFA, ESR1, MYC, MAPK1, and PPARG, may play critical roles in the therapeutic effects of WLHP on lactation insufficiency owing to their high connectivity within the network and their involvement in crucial biological processes related to lactation.

### 3.10 Functional enrichment of overlapping WLHP-lactation targets

**FIGURE 7:**
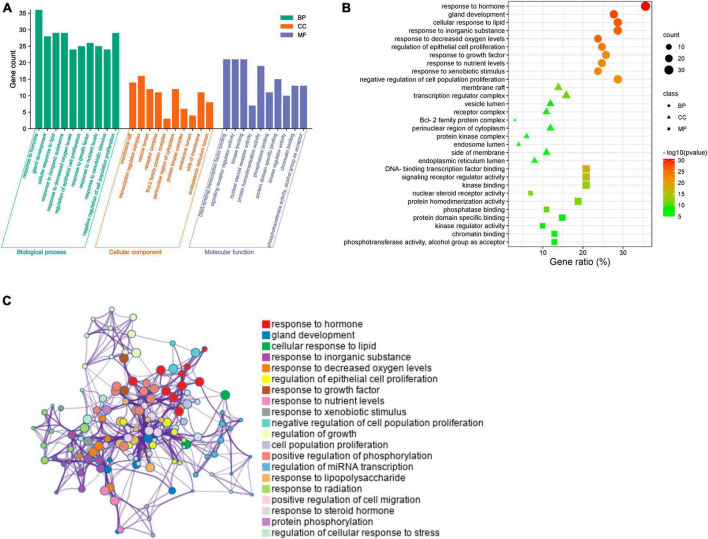
Functional enrichment analysis of overlapping targets between WLHP and lactation. **(A)** Bar chart displaying the top 10 enriched biological process (BP), cellular component (CC), and molecular function (MF) terms. **(B)** Bubble diagram representing the top 10 enriched gene ontology (GO) terms based on -log10 (*P*-value). **(C)** Network diagram showcasing the interrelationships among the top 20 representative BP terms. node sizes, colors, and edge thicknesses indicate the number of input genes, cluster identity, and term similarity, respectively.

To further investigate the mechanisms underlying the 101 overlapping targets between WLHP and lactation, we performed a comprehensive functional enrichment analysis, including GO functional annotation and KEGG pathway enrichment, using the Metascape platform. Terms with a *p*-value < 0.01, a minimum count of 3, and an enrichment factor > 1.5 were considered statistically significant. The top 10 GO terms provided a deeper understanding of the key BPs, CCs, and MFs associated with the potential effects of WLHP on lactation (Figures 7A, B). The key enriched BP terms related to lactation included response to hormones, gland development, cellular response to lipids, regulation of epithelial cell proliferation, response to growth factors, and response to nutrient levels. To better understand the relationships among the BP terms, we selected the 20 top representative terms and converted them into a network layout (Figure 7C). In this network, each node represents a BP term and its size corresponds to the number of genes belonging to that term. The node color indicates its cluster, meaning that nodes of the same color belong to the same cluster. Nodes are linked if they share a similarity score > 0.3, with the thickness of the link (edge) representing the magnitude of the score.

**FIGURE 8:**
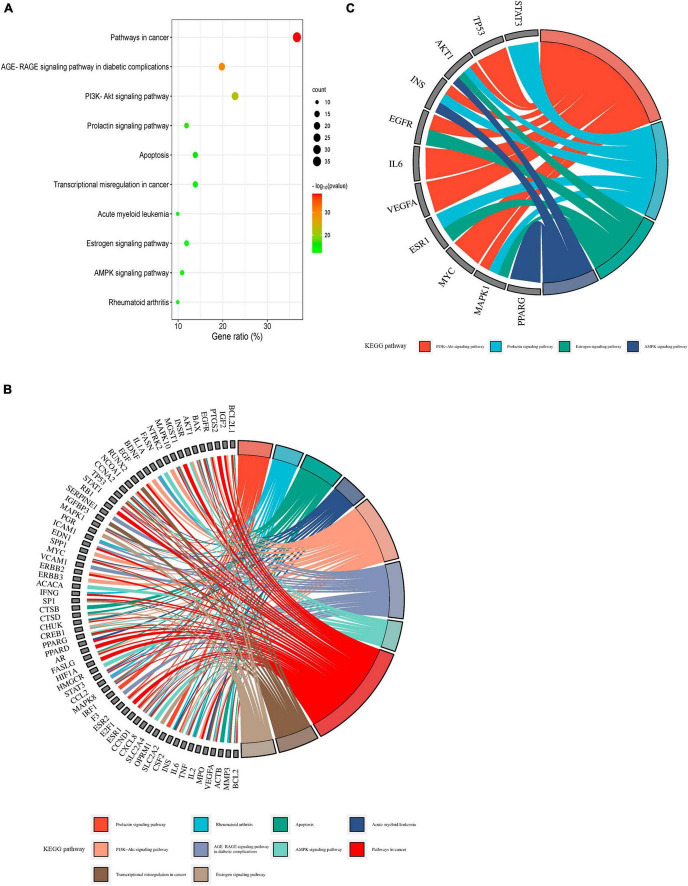
Comprehensive Kyoto Encyclopedia of Genes and Genomes (KEGG) pathway enrichment analysis of overlapping targets in WLHP-lactation. **(A)** Bar chart of the top 10 KEGG pathways enriched in WLHP-lactation overlapping targets. **(B)** Chord diagram depicting the interrelationships between overlapping targets and the top 10 KEGG pathways. **(C)** Specific interplay between hub genes and the four key pathways (PI3K-Akt signaling pathway, Prolactin signaling pathway, Estrogen signaling pathway, and AMPK signaling pathway) represented as a chord diagram.

**FIGURE 9:**
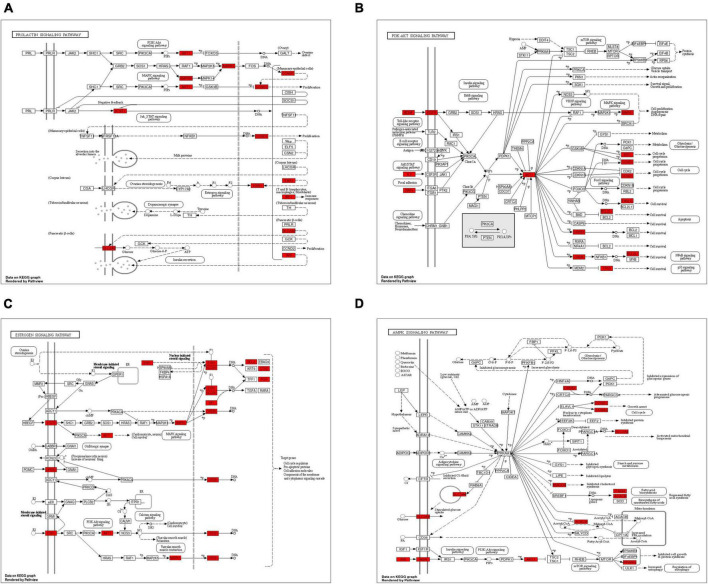
Detailed visualization of overlapping targets within major Kyoto Encyclopedia of Genes and Genomes (KEGG) pathways involved in WLHP’s potential therapeutic mechanisms for lactation insufficiency. **(A)** PI3K-Akt signaling pathway. **(B)** Prolactin signaling pathway. **(C)** Estrogen signaling pathway. **(D)** AMPK signaling pathway. Each subfigure represents the respective pathway, with overlapping targets highlighted to illustrate their distribution and involvement in these signaling pathways.

The top 10 KEGG pathways highlight the critical signaling pathways possibly involved in the mechanisms through which WLHP exerts its therapeutic influence on lactation regulation (Figure 8A). The relationship between the overlapping targets of WLHP-lactation and the top 10 KEGG pathways is illustrated in Figure 8B using a chord diagram. In this diagram, overlapping targets are displayed on the left side, whereas the top 10 KEGG pathways are positioned on the right side. Colored chords connect each target with its respective pathway, effectively representing the complex interconnections and potential regulatory roles of these targets in the therapeutic effects of WLHP on lactation insufficiency. Among the top 10 most enriched signaling pathways, we further combined a comprehensive literature search to identify four key pathways, including the PI3K-Akt signaling pathway, prolactin signaling pathway, estrogen signaling pathway, and AMPK signaling pathway. To better understand the interactions and relationships between the overlapping targets of WLHP-lactation and their corresponding KEGG pathways, we generated diagrams for each of the four KEGG pathways. These visualizations effectively illustrate the distribution and involvement of these overlapping targets within the key signaling pathways, providing valuable insights into the potential therapeutic mechanisms of WLHP in lactation insufficiency (Figures 9A–D). The identified hub genes serve as key elements in regulating the WLHP-lactation relationship. Lactation regulation involves several crucial pathways, including the PI3K-Akt, prolactin, estrogen, and AMPK signaling pathways. To illustrate the connections between the hub genes and these four pathways, we used a chord diagram, which effectively represented the interplay between these essential components in the context of the potential therapeutic effects of WLHP on lactation insufficiency (Figure 8C). In summary, our findings elucidate the complex relationships among herbs, key potent compounds, regulated hub genes, and lactation-related pathways involved in WLHP for lactation insufficiency. For a more detailed overview of these relationships, Table 6 presents a comprehensive summary of these key elements.

**TABLE 6:** Summary of herbs, key potent compounds, regulated hub genes, and involved lactation-related pathways in WLHP for lactation insufficiency.

	Herbs	Key potent compounds	Regulated hub genes	Involved lactation-related pathways
WLHP	Wang Bu Liu Xing	Quercetin	EGFR, AKT1, VEGFA, MAPK1, TNF, IL6, TP53, PPARG, MYC	PI3K-Akt signaling pathway, Prolactin signaling pathway, Estrogen signaling pathway, and AMPK signaling pathway
		Oleic acid	PPARG, INS	PI3K-Akt signaling pathway, Prolactin signaling pathway, Estrogen signaling pathway, and AMPK signaling pathway
	Lu Lu Tong	Ursolic acid	STAT3, VEGFA, TNF, IL6, TP53	PI3K-Akt signaling pathway, Prolactin signaling pathway

## 4 Discussion

This study comprehensively analyzed the use of CHP among patients with lactation insufficiency and examined the underlying molecular mechanisms through which CHP, particularly the WLHP combination, might exert therapeutic effects. Detailed evaluation of CHP prescription patterns, demographic characteristics, co-existing diseases, potential compounds, and targets provided insights crucial to our understanding regarding the role of CHP in managing lactation insufficiency.

In this study, we included 490 patients with lactation insufficiency. However, this number may not reflect the full scale of the issue. There could be many individuals dealing with lactation insufficiency who opt for self-treatment strategies, such as home remedies, dietary adjustments, or over-the-counter CHP without professional medical consultation. These cases remain largely unreported, contributing to underestimation of the true prevalence of lactation insufficiency. Among these, our results underscore a profound inclination toward the use of CHP among patients diagnosed with lactation insufficiency in Taiwan, with a striking majority of > 80% seeking CHP treatment. This prominent preference emphasizes a deep-rooted faith in the effectiveness of CHP for managing lactation insufficiency, thus elucidating its cultural and therapeutic significance in this population. This finding aligns with the cultural and historical prevalence of TCM in the country, where it has been deeply ingrained in healthcare practices for centuries. This usage pattern also supports the findings of previous studies noting the widespread use of CHP for various conditions within the Taiwanese population (27, 28). Our research demonstrates that most patients with lactation insufficiency have utilized the widely prescribed pharmacological galactagogues, including metoclopramide and domperidone. Domperidone was the most commonly prescribed medication among both CHP and non-CHP users. Based on these findings, the observed predilection toward CHP use could indicate possible dissatisfaction or a lack of complete resolution of lactation issues using conventional medical treatments. This hypothesis is congruent with studies underscoring that patients often gravitate toward TCM and other complementary therapies when conventional medicine fails to satisfactorily address their health concerns (29, 30). Consequently, despite the widespread use of standard pharmacological galactagogues, a substantial inclination for alternative or supplementary solutions, such as CHP, is seen among the patient population dealing with lactation insufficiency in Taiwan.

Despite the broad coverage of Taiwan’s health insurance, over-the-counter CHP is not recorded in the healthcare database. Interestingly, a study conducted on 127 postpartum women revealed that 21.3% (*n* = 27) sought herbal remedies from clinics, while a striking 63.0% (*n* = 80) opted for pharmacies as their primary source of herbal treatments (31). These data suggest that, beyond formal healthcare facilities, pharmacies play a pivotal role in providing access to CHP for lactation insufficiency. A thorough investigation into the use of medicinal materials for inducing lactation in TCM revealed a collection of 81 different substances from 87 TCM pharmacies across Taiwan. Among these, 19 materials were frequently cited, and Dang Gui emerged as the primary core medicinal material (22). Although this study holds considerable value, the large proportion of individuals purchasing medication from pharmacies highlights a discrepancy in the prescription patterns observed in our study. This considerable reliance on CHP raises significant questions regarding safety and quality control, particularly when self-administered without professional guidance. We argue that the prescription models provided by well-trained Taiwanese TCM practitioners are invaluable as references. These practitioners, grounded in the unique healthcare context of Taiwan, seamlessly combine traditional theories with modern knowledge in physiology and pharmacology to provide comprehensive and personalized healthcare services to their patients. Therefore, utilization of the NHIRD for this study has substantial significance. The NHIRD captures a detailed record of prescriptions issued by professionally trained TCM doctors, offering an accurate reflection of TCM utilization in Taiwan’s healthcare landscape. In Taiwan’s health insurance system, TCM doctors primarily prescribe concentrated herbal extract granules, which offer several benefits. The use of concentrated herbal extracts ensures consistent potency and quality of herbs as well as simplifies the preparation and administration process for patients. This approach is more convenient and time-saving than traditional decoctions, making it an attractive option for modern lifestyles. Furthermore, the unique prescription mode of concentrated herbal extracts allows the flexible use of both Chinese herbal formulas and single Chinese herbs. TCM doctors can create customized treatment plans based on individual patient needs, symptoms, and constitutions by combining or adjusting the dosages of various concentrated herbal extracts.

Breastfeeding, while providing numerous benefits to both the mother and infant, can be physically challenging owing to the need for prolonged and sometimes awkward postures, potentially leading to musculoskeletal discomfort, such as joint pain (32, 33). As highlighted in our study, we found that many women with lactation insufficiency, particularly those not using CHP, reported musculoskeletal and joint pain as their primary coexisting condition. Further, the diverse range of common symptoms reported by breastfeeding mothers such as headache, dyspepsia, insomnia, and other discomforts (34–36), further underscores the comprehensive approach adopted by TCM doctors. Rather than simply treating lactation insufficiency, they address multiple concurrent health issues that can significantly affect the overall wellbeing of nursing mothers. The multifaceted approach of TCM, focusing on the whole person, provides considerable benefits for breastfeeding mothers, who often face various physical discomforts during the postpartum period. This highlights the unique role that TCM can play in providing holistic care and support to postpartum women.

Chinese herbal formulas, composed of multiple medicinal substances, can consider multiple disease factors or the diverse range of symptoms accompanying the disease. In the context of lactation insufficiency, our results indicate that Jia Wei Xiao Yao San, Ba Zhen Tang, and Gui Pi Tang are the most commonly used formulas. Lactation is a complex physiological process governed by a delicate interplay between hormones and environmental factors. While hormones such as prolactin and oxytocin are essential for milk production and ejection, factors such as stress, fatigue, and mental wellbeing also influence this process significantly (37, 38). The physical and emotional changes a woman undergoes during the postpartum phase can further compound these challenges, potentially leading to lactation insufficiency. Jia Wei Xiao Yao San, recognized for its calming effects, may alleviate stress (39), a potential contributor to lactation insufficiency. Traditionally, it is believed to harmonize the body systems, an approach that may prove beneficial during periods of hormonal fluctuations, such as the pregnant and postpartum phases (40, 41). Ba Zhen Tang, known for its tonifying properties, is considered helpful in restoring energy and managing fatigue (42). Frequently used to enhance general wellbeing and post-labor recovery, it indirectly supports breastfeeding by boosting maternal physical health. Gui Pi Tang, traditionally employed in TCM to bolster the spleen and nourish the heart, may help address sleep disturbances and mental distress (43, 44), which could potentially affect lactation.

Among the various single Chinese herbs used in TCM, Wang Bu Liu Xing and Lu Lu Tong have emerged as the most frequently prescribed for lactation insufficiency. These herbs have been commonly prescribed to support lactation in TCM. Wang Bu Liu Xing is traditionally used for promoting lactation and treating breast abscess (45), while Lu Lu Tong has been linked with improving circulation and reducing swelling (46). Our study further revealed that among dual combinations of CHP, the pairing of Wang Bu Liu Xing and Lu Lu Tong was the most common. The correlation network graph, which further represented the relationships between various single Chinese herbs and Chinese herbal formulas used for treating lactation insufficiency, placed the WLHP combination at its core. Herb pairs are crucial in TCM because they work synergistically to enhance treatment efficacy, minimize side effects, and harmonize the actions of each herb (47, 48). The prominence of this particular herb pair, referred to as WLHP in this study, underscores its importance in treating lactation insufficiency.

We identified key potent compounds in Wang Bu Liu Xing and Lu Lu Tong, including quercetin, oleic acid, and ursolic acid, which were connected to multiple overlapping WLHP-lactation targets. A study has suggested that quercetin, a natural phytoestrogen, can enhance lactation and mammary gland development in mice with agalactosis, likely by stimulating prolactin production and release, promoting mammary epithelial cell proliferation, and increasing the expression of key proteins involved in milk production (49). Oleic acid, a monounsaturated fatty acid, suppresses overexpression of the Her-2/neu oncogene, which is associated with breast cancer progression, and enhances the efficacy of anti-Her-2/neu immunotherapy, contributing to apoptotic cell death in breast cancer cells (50). A previous study found that ursolic acid exerts anti-cancer effects on breast cancer cells by inducing autophagy and apoptosis, suppressing cell invasiveness, and inhibiting inflammation via the PI3K/AKT, GSK, caspase-3, and NF-κB signaling pathways (51). Considering the impact of oleic acid and ursolic acid on breast tissue, further research should investigate their potential application in promoting lactation, as breast health is central to successful breastfeeding. Interestingly, a study conducted on Lu Lu Tong isolates identified 13 triterpenoids with potential anti-breast cancer effects, via inhibition of multiple protein targets such as ErbB4 and EGFR. However, researchers have questioned its traditional use for lactation enhancement owing to its known inhibitory effect on ErbB4, which can theoretically suppress lactation (52). Contrary to this conclusion, our research found significant usage of Lu Lu Tong in Taiwanese TCM for managing lactation insufficiency, indicating its perceived effectiveness in promoting lactation. It is crucial to understand that TCM relies on a complex interplay of multiple compounds, working synergistically, which might not be reflected in isolated compound studies. The overall effect of a medicinal plant cannot be reduced to the action of a single component. The application and dosage of TCM are based on a holistic understanding of the patient condition, which might modulate the effects of individual components. Consequently, caution should be exercised before discarding the established therapeutic uses of such traditional remedies based on studies of isolated compounds.

PPI network and topology analysis led us to identify 13 potential hub genes associated with lactation: STAT3, TP53, AKT1, TNF, ACTB, INS, EGFR, IL6, VEGFA, ESR1, MYC, MAPK1, and PPARG. For instance, a study demonstrated that the levels of epidermal growth factor (EGF) and transforming growth factor-α (TGF-α), which are important growth-promoting factors in breast milk, change across lactation stages and are influenced by geographical location and maternal diet (53). Another study reported that during the first 12 weeks of lactation, the concentrations of proinflammatory cytokines IL-1β, IL-6, TNF-α, and anti-inflammatory cytokines TGF-β1, TGF-β2 in human milk vary along with prostaglandin E2. Proinflammatory cytokines were present only in a subset of samples, whereas anti-inflammatory cytokines were consistently found in substantial quantities. These findings suggest a significant role for these immunomodulatory agents in infant health during early breastfeeding (54). Furthermore, the essential amino acid l-Lysine (l-Lys) was found to enhance milk protein synthesis in dairy cow mammary epithelial cells. This was achieved by upregulating proteins, including MAPK1, which stimulates milk protein production through the Stat5 and mTOR pathways, signifying a critical role for MAPK1 in regulating milk protein synthesis (55).

The PI3K-Akt, prolactin, estrogen, and AMPK signaling pathways have been identified as integral to the therapeutic mechanisms of WLHP in addressing lactation insufficiency. One study specifically examined the effect of lauric acid (LA) on pubertal mammary gland development in mice. The results demonstrated that LA enhances the proliferation of mammary epithelial cells and mammary duct development. Additionally, it was shown to upregulate the expression of proliferative markers and G protein-coupled receptor 84 (GPR84). Notably, these outcomes were mediated by activation of the PI3K/Akt signaling pathway (56). Prolactin, an anterior pituitary-secreted hormone, is essential for establishing and maintaining lactation, regulating milk macronutrient contents, and stimulating milk production. During pregnancy, prolactin levels rise dramatically and initiate extensive milk secretion upon the postpartum clearance of progesterone and estrogen. Its vital functions include promoting and stabilizing casein mRNA transcription, potentially enhancing alpha-lactalbumin synthesis, increasing mammary gland lipoprotein lipase activity, and sustaining milk production through the release induced by nursing (57). This study explored changes in the mRNA expression of estrogen receptor subtypes (ERα, ERβ, and TERP-1) within the rat pituitary gland across the gestation, lactation, and post-lactation periods. ERα mRNA was found to peak mid-pregnancy while maintaining constant protein levels, while ERβ levels remained stable, and TERP-1 levels increased significantly in late gestation, suggesting the potential critical role of TERP-1 in reproductive events (58). This study investigated the potential epigenetic mechanisms related to breast cancer onset through reproductive risk factors by examining methylation alterations at the CpG island promoter of the estrogen receptor β (ER-β) gene in healthy women. The results indicated a higher level of ER-β exon 0 N methylation in nulliparous and non-breastfeeding women, suggesting that changes in methylation may be linked with reproductive history. However, no significant relationship was observed between methylation at promoter 0 N and reproductive history (59). Another study revealed the significant role of AMP-activated protein kinase (AMPK) in the synthesis of milk fat and protein, particularly under energy-deficient conditions. AMPK was found to regulate milk protein synthesis primarily through the prolactin signaling pathway, leading to degradation of the prolactin receptor (PrlR). Moreover, it was shown to influence milk fat synthesis by inhibiting *de novo* fatty acid synthesis and promoting fatty acid oxidation. These results suggest that regulation of milk synthesis by AMPK is not solely dependent on mTORC1 signaling, but also involves modulation of PrlR degradation and PGC-1α acetylation (60).

Our study, while comprehensive, has several limitations. First, the database used to identify patients with lactation insufficiency relied solely on physician-recorded diagnoses. This methodology could potentially introduce selection bias and limit the generalizability of our findings, as the sample might not reflect all mothers with lactation insufficiency, particularly those who have not sought medical help. Second, the study utilized cross-sectional data, precluding the determination of cause-effect relationships between CHP usage and lactation insufficiency. Third, our results were derived from a Taiwanese database, potentially limiting their generalizability to other ethnic or geographic populations. Future studies should address these limitations and validate our findings using prospective designs and more diverse populations. Furthermore, while the TCMSP database is a comprehensive resource, it may not encompass all potential compounds and targets found in the herbs under study. Additionally, *in silico* bioinformatics analyses rely on databases that may inherently contain biases shaped by their included literature. Finally, our research employed bioinformatics analysis, a potent method yet one that may yield primarily associative results. Therefore, future experimental validation of our bioinformatics findings is essential to substantiate the mechanisms and therapeutic effects of WLHP in addressing lactation insufficiency. Despite these limitations, our study provides a crucial step forward in understanding the potential therapeutic effects of CHP on lactation insufficiency and paves the way for future research.

## 5 Conclusion

In conclusion, our study provides substantial insights into the prevalence of CHP use to address lactation insufficiency. Through an in-depth examination of prescription patterns, we discovered that the combination of Wang Bu Liu Xing and Lu Lu Tong, known as WLHP, is often used as a treatment strategy. The biological relevance of WLHP was further corroborated by our bioinformatics analyses, which uncovered an intricate interplay between various compounds, targets, and key signaling pathways. This finding resonates with the core philosophy of Chinese Medicine that advocates a holistic, multi-compound, and multi-target treatment approach. Our study successfully merges traditional medicinal wisdom with contemporary scientific methodologies, thus fostering a greater understanding of the potential role of CHP and setting the stage for further investigations into innovative treatment modalities for lactation insufficiency.

## Data availability statement

The original contributions presented in this study are included in this article/supplementary material, further inquiries can be directed to the corresponding author.

## Ethics statement

The studies involving humans were approved by the Institutional Review Board of the China Medical University in Central Taiwan (CMUH111-REC2-109). The studies were conducted in accordance with the local legislation and institutional requirements. The Ethics Committee/Institutional Review Board waived the requirement of written informed consent for participation from the participants or the participants’ legal guardians/next of kin because the study uses de-identified data, protecting individual privacy and facilitating health research.

## Author contributions

C-CL: Conceptualization, Data curation, Formal analysis, Methodology, Validation, Visualization, Writing—original draft. C-HC: Formal analysis, Investigation, Methodology, Writing—original draft. T-JH: Data curation, Formal analysis, Investigation, Software, Visualization, Writing—original draft. J-ML: Conceptualization, Funding acquisition, Project administration, Supervision, Validation, Writing—review and editing.

## Footnotes

1
https://old.tcmsp-e.com

2
https://www.uniprot.org

3
https://omim.org

4
https://www.disgenet.org

5
https://www.genecards.org

6
https://bioinfogp.cnb.csic.es/tools/venny/

7
https://string-db.org

8
https://metascape.org

## References

[1] Kim SY Yi DY. Components of human breast milk: from macronutrient to microbiome and microRNA. *Clin Exp Pediatr.* (2020) 63:301–9. 10.3345/cep.2020.00059 32252145 PMC7402982

[2] Ballard O Morrow AL. Human milk composition: nutrients and bioactive factors. *Pediatr Clin North Am.* (2013) 60:49–74. 10.1016/j.pcl.2012.10.002 23178060 PMC3586783

[3] Walker WA Iyengar RS. Breast milk, microbiota, and intestinal immune homeostasis. *Pediatr Res.* (2015) 77:220–8. 10.1038/pr.2014.160 25310762

[4] Cacho NT Lawrence RM. Innate immunity and breast milk. *Front Immunol.* (2017) 8:584. 10.3389/fimmu.2017.00584 28611768 PMC5447027

[5] Perrella S Gridneva Z Lai CT Stinson L George A Bilston-John S Human milk composition promotes optimal infant growth, development and health. *Semin Perinatol.* (2021) 45:151380. 10.1016/j.semperi.2020.151380 33431112

[6] Martin CR Ling PR Blackburn GL. Review of infant feeding: key features of breast milk and infant formula. *Nutrients.* (2016) 8:279. 10.3390/nu8050279 27187450 PMC4882692

[7] Donald K Petersen C Turvey SE Finlay BB Azad MB. Secretory IgA: linking microbes, maternal health, and infant health through human milk. *Cell Host Microbe.* (2022) 30:650–9. 10.1016/j.chom.2022.02.005 35550668

[8] Tucker Z O’Malley C. Mental health benefits of breastfeeding: a literature review. *Cureus.* (2022) 14:e29199. 10.7759/cureus.29199 36258949 PMC9572809

[9] Krol KM Grossmann T. Psychological effects of breastfeeding on children and mothers. *Bundesgesundheitsblatt Gesundheitsforschung Gesundheitsschutz.* (2018) 61:977–85. 10.1007/s00103-018-2769-0 29934681 PMC6096620

[10] Kramer MS Kakuma R. Optimal duration of exclusive breastfeeding. *Cochr Database Syst Rev.* (2012) 2012:Cd003517. 10.1002/14651858.CD003517.pub2 22895934 PMC7154583

[11] Gatti L. Maternal perceptions of insufficient milk supply in breastfeeding. *J Nurs Scholarsh.* (2008) 40:355–63. 10.1111/j.1547-5069.2008.00234.x 19094151 PMC4508856

[12] Gökçeoğlu E Küçükoğlu S. The relationship between insufficient milk perception and breastfeeding self-efficacy among Turkish mothers. *Glob Health Promot.* (2017) 24:53–61. 10.1177/1757975916635080 27353118

[13] Hill PD Aldag JC Demirtas H Naeem V Parker NP Zinaman MJ Association of serum prolactin and oxytocin with milk production in mothers of preterm and term infants. *Biol Res Nurs.* (2009) 10:340–9. 10.1177/1099800409331394 19224938

[14] Farah E Barger MK Klima C Rossman B Hershberger P. Impaired lactation: review of delayed lactogenesis and insufficient lactation. *J Midwifery Womens Health.* (2021) 66:631–40. 10.1111/jmwh.13274 34596953

[15] Motee A Ramasawmy D Pugo-Gunsam P Jeewon R. An assessment of the breastfeeding practices and infant feeding pattern among mothers in mauritius. *J Nutr Metab.* (2013) 2013:243852. 10.1155/2013/243852 23864943 PMC3707234

[16] McBride GM Stevenson R Zizzo G Rumbold AR Amir LH Keir AK Use and experiences of galactagogues while breastfeeding among Australian women. *PLoS One.* (2021) 16:e0254049. 10.1371/journal.pone.0254049 34197558 PMC8248610

[17] Shen Q Khan KS Du MC Du WW Ouyang YQ. Efficacy and safety of domperidone and metoclopramide in breastfeeding: a systematic review and meta-analysis. *Breastfeed Med.* (2021) 16:516–29. 10.1089/bfm.2020.0360 33769844

[18] Paul C Zénut M Dorut A Coudoré M-A Vein J Cardot J-M Use of domperidone as a galactagogue drug: a systematic review of the benefit-risk ratio. *J Hum Lact.* (2015) 31:57–63. 10.1177/0890334414561265 25475074

[19] Zizzo G Rumbold AR Grzeskowiak LE. “Fear of stopping” vs “wanting to get off the medication”: exploring women’s experiences of using domperidone as a galactagogue - a qualitative study. *Int Breastfeed J.* (2021) 16:92. 10.1186/s13006-021-00438-5 34886887 PMC8656031

[20] Sim TF Hattingh HL Sherriff J Tee LB. The Use, Perceived effectiveness and safety of herbal galactagogues during breastfeeding: a qualitative study. *Int J Environ Res Public Health.* (2015) 12:11050–71. 10.3390/ijerph120911050 26371019 PMC4586661

[21] Budzynska K Gardner ZE Low Dog T Gardiner P. Complementary, holistic, and integrative medicine: advice for clinicians on herbs and breastfeeding. *Pediatr Rev.* (2013) 34:343–52; quiz352–3. 10.1542/pir.34-8-343 23908361 PMC4530286

[22] Chao J Ko CY Lin CY Tomoji M Huang CH Chiang HC Ethnobotanical survey of natural galactagogues prescribed in traditional Chinese medicine pharmacies in Taiwan. *Front Pharmacol.* (2020) 11:625869. 10.3389/fphar.2020.625869 33679390 PMC7928277

[23] Zhang WB Wang GJ Fuxe K. Classic and modern meridian studies: a review of low hydraulic resistance channels along Meridians and their relevance for therapeutic effects in traditional Chinese medicine. *Evid Based Complement Alternat Med.* (2015) 2015:410979. 10.1155/2015/410979 25821487 PMC4363694

[24] Klein P Picard G Baumgarden J Schneider R. Meditative movement, energetic, and physical analyses of three Qigong exercises: unification of Eastern and Western mechanistic exercise theory. *Medicines.* (2017) 4:69. 10.3390/medicines4040069 28946612 PMC5750593

[25] Chuang CH Chang PJ Hsieh WS Tsai YJ Lin SJ Chen PC. Chinese herbal medicine use in Taiwan during pregnancy and the postpartum period: a population-based cohort study. *Int J Nurs Stud.* (2009) 46:787–95. 10.1016/j.ijnurstu.2008.12.015 19193377

[26] Hsieh CY Su CC Shao SC Sung SF Lin SJ Kao Yang YH Taiwan’s national health insurance research database: past and future. *Clin Epidemiol.* (2019) 11:349–58. 10.2147/clep.S196293 31118821 PMC6509937

[27] Lai JN Wu CT Wang JD. Prescription pattern of Chinese herbal products for breast cancer in Taiwan: a population-based study. *Evid Based Complement Alternat Med.* (2012) 2012:891893. 10.1155/2012/891893 22685488 PMC3368194

[28] Chen MC Lai JN Chen PC Wang JD. Concurrent use of conventional drugs with Chinese herbal products in Taiwan: a population-based study. *J Tradit Complement Med.* (2013) 3:256–62. 10.4103/2225-4110.119734 24716186 PMC3925000

[29] Lu AP Jia HW Xiao C Lu QP. Theory of traditional Chinese medicine and therapeutic method of diseases. *World J Gastroenterol.* (2004) 10:1854–6. 10.3748/wjg.v10.i13.1854 15222022 PMC4572216

[30] Zhang X Qiu H Li C Cai P Qi F. The positive role of traditional Chinese medicine as an adjunctive therapy for cancer. *Biosci Trends.* (2021) 15:283–98. 10.5582/bst.2021.01318 34421064

[31] Ho M Li T-C Su S-Y. The association between traditional Chinese dietary and herbal therapies and uterine involution in postpartum women. *Evid Based Complement Alternat Med.* (2011) 2011:918291. 10.1155/2011/918291 21584195 PMC3092725

[32] Aburub A Darabseh MZ Alsharman A Hegazy MM Hunter SM. Nursing mothers’ experiences of musculoskeletal pain attributed to poor posture during breastfeeding: a mixed methods study. *Breastfeed Med.* (2022) 17:926–31. 10.1089/bfm.2022.0105 36378819

[33] Rani S Habiba UE Qazi WA Tassadaq N. Association of breast feeding positioning with musculoskeletal pain in post partum mothers of Rawalpindi and Islamabad. *J Pak Med Assoc.* (2019) 69:564–6. 31000863

[34] Wall VR. Breastfeeding and migraine headaches. *J Hum Lact.* (1992) 8:209–12. 10.1177/089033449200800422 1288557

[35] Thélin CS Richter JE. Review article: the management of heartburn during pregnancy and lactation. *Aliment Pharmacol Ther.* (2020) 51:421–34. 10.1111/apt.15611 31950535

[36] Ko YL Lin SC Lin PC. Effect of auricular acupressure for postpartum insomnia: an uncontrolled clinical trial. *J Clin Nurs.* (2016) 25:332–9. 10.1111/jocn.13053 26612319

[37] Nagel EM Howland MA Pando C Stang J Mason SM Fields DA Maternal psychological distress and lactation and breastfeeding outcomes: a narrative review. *Clin Ther.* (2022) 44:215–27. 10.1016/j.clinthera.2021.11.007 34937662 PMC8960332

[38] Keith DR Weaver BS Vogel RL. The effect of music-based listening interventions on the volume, fat content, and caloric content of breast milk-produced by mothers of premature and critically ill infants. *Adv Neonatal Care.* (2012) 12:112–9. 10.1097/ANC.0b013e31824d9842 22469966

[39] Ji S Han S Yu L Du L You Y Chen J Jia Wei Xiao Yao San ameliorates chronic stress-induced depression-like behaviors in mice by regulating the gut microbiome and brain metabolome in relation to purine metabolism. *Phytomedicine.* (2022) 98:153940. 10.1016/j.phymed.2022.153940 35104765

[40] Li Y Chen Z Yu N Yao K Che Y Xi Y Chinese herbal medicine for postpartum depression: a systematic review of randomized controlled trials. *Evid Based Complement Alternat Med.* (2016) 2016:5284234. 10.1155/2016/5284234 27774110 PMC5059536

[41] Wen SH Chang WC Shen HS Wu HC. Prescription patterns and factors influencing the use of Chinese herbal medicine among pregnant women in Taiwan: a population-based retrospective study. *BMC Complement Med Ther.* (2020) 20:240. 10.1186/s12906-020-03032-0 32731888 PMC7391530

[42] Han M Li H Ke D Tian LM Hong Y Zhang C Mechanism of Ba Zhen Tang delaying skin photoaging based on network pharmacology and molecular docking. *Clin Cosmet Investig Dermatol.* (2022) 15:763–81. 10.2147/ccid.S344138 35510223 PMC9058032

[43] Ni X Shergis JL Zhang AL Guo X Lu C Li Y Traditional use of Chinese Herbal Medicine for insomnia and priorities setting of future clinical research. *J Altern Complement Med.* (2019) 25:8–15. 10.1089/acm.2018.0249 30376350

[44] Liu L Liu C Wang Y Wang P Li Y Li B. Herbal medicine for anxiety, depression and insomnia. *Curr Neuropharmacol.* (2015) 13:481–93. 10.2174/1570159x1304150831122734 26412068 PMC4790408

[45] Chou SH Huang CC Lin CH Wu KC Chiang PJ. General Use of Chinese herbal products among Female Patients with Mastitis in Taiwan. *Evid Based Complement Alternat Med.* (2022) 2022:3876240. 10.1155/2022/3876240 35368771 PMC8975662

[46] Li WX Qian P Guo YT Gu L Jurat J Bai Y Myrtenal and β-caryophyllene oxide screened from Liquidambaris Fructus suppress NLRP3 inflammasome components in rheumatoid arthritis. *BMC Complement Med Ther.* (2021) 21:242. 10.1186/s12906-021-03410-2 34583676 PMC8480017

[47] Hu XQ Sun Y Lau E Zhao M Su SB. Advances in synergistic combinations of Chinese herbal medicine for the treatment of cancer. *Curr Cancer Drug Targets.* (2016) 16:346–56. 10.2174/1568009616666151207105851 26638885 PMC5425653

[48] Che CT Wang ZJ Chow MS Lam CW. Herb-herb combination for therapeutic enhancement and advancement: theory, practice and future perspectives. *Molecules.* (2013) 18:5125–41. 10.3390/molecules18055125 23644978 PMC6269890

[49] Lin M Wang N Yao B Zhong Y Lin Y You T. Quercetin improves postpartum hypogalactia in milk-deficient mice via stimulating prolactin production in pituitary gland. *Phytother Res.* (2018) 32:1511–20. 10.1002/ptr.6079 29671937

[50] Menendez JA Vellon L Colomer R Lupu R. Oleic acid, the main monounsaturated fatty acid of olive oil, suppresses Her-2/neu (erbB-2) expression and synergistically enhances the growth inhibitory effects of trastuzumab (Herceptin) in breast cancer cells with Her-2/neu oncogene amplification. *Ann Oncol.* (2005) 16:359–71. 10.1093/annonc/mdi090 15642702

[51] Luo J Hu YL Wang H. Ursolic acid inhibits breast cancer growth by inhibiting proliferation, inducing autophagy and apoptosis, and suppressing inflammatory responses via the PI3K/AKT and NF-κB signaling pathways in vitro. *Exp Ther Med.* (2017) 14:3623–31. 10.3892/etm.2017.4965 29042957 PMC5639319

[52] Qian P Mu X-T Su B Gao L Zhang D-F. Identification of the anti-breast cancer targets of triterpenoids in Liquidambaris Fructus and the hints for its traditional applications. *BMC Complement Med Ther.* (2020) 20:369. 10.1186/s12906-020-03143-8 33246450 PMC7694930

[53] Lu M Jiang J Wu K Li D. Epidermal growth factor and transforming growth factor-α in human milk of different lactation stages and different regions and their relationship with maternal diet. *Food Funct.* (2018) 9:1199–204. 10.1039/c7fo00770a 29379938

[54] Hawkes JS Bryan DL James MJ Gibson RA. Cytokines (IL-1beta, IL-6, TNF-alpha, TGF-beta1, and TGF-beta2) and prostaglandin E2 in human milk during the first three months postpartum. *Pediatr Res.* (1999) 46:194–9. 10.1203/00006450-199908000-00012 10447115

[55] Lu LM Li QZ Huang JG Gao XJ. Proteomic and functional analyses reveal MAPK1 regulates milk protein synthesis. *Molecules.* (2012) 18:263–75. 10.3390/molecules18010263 23271465 PMC6270553

[56] Meng Y Zhang J Zhang F Ai W Zhu X Shu G Lauric acid stimulates mammary gland development of pubertal mice through activation of GPR84 and PI3K/Akt signaling pathway. *J Agric Food Chem.* (2017) 65:95–103. 10.1021/acs.jafc.6b04878 27978622

[57] Ostrom KM. A review of the hormone prolactin during lactation. *Prog Food Nutr Sci* (1990) 14:1–43. 2092340

[58] Vaillant C Chesnel F Schausi D Tiffoche C Thieulant M-L. Expression of estrogen receptor subtypes in rat pituitary gland during pregnancy and lactation. *Endocrinology.* (2002) 143:4249–58. 10.1210/en.2002-220193 12399419

[59] Daraei A Izadi P Khorasani G Nafissi N Naghizadeh MM Meysamie A A methylation signature at the CpG island promoter of estrogen receptor beta (ER-β) in breasts of women may be an early footmark of lack of breastfeeding and nulliparity. *Pathol Res Pract.* (2021) 218:153328. 10.1016/j.prp.2020.153328 33422777

[60] Wu Z Li Q Yang S Zheng T Shao J Guan W Energy deprivation-induced AMPK activation inhibits milk synthesis by targeting PrlR and PGC-1α. *Cell Commun Signal.* (2022) 20:25. 10.1186/s12964-022-00830-6 35248054 PMC8898430

